# Targeting AATF reprograms the tumor microenvironment and suppresses hepatocellular carcinoma via MIR100HG-TGF-β signaling

**DOI:** 10.1016/j.omton.2026.201328

**Published:** 2026-08-21

**Authors:** Diwakar Suresh, Akshatha N. Srinivas, Bharathwaaj Gunaseelan, S.A. Amith Bharadwaj, Manju Moorthy, Gopalkrishna Ramaswamy, Suchitha Satish, Prashant Vishwanath, Prasanna Kumar Santhekadur, Saravana Babu Chidambaram, Divya P. Kumar

## Main text

(Molecular Therapy: Oncology *34*, 1–17; September 2026)

In the originally published version of this article, Figure 1C, demonstrating tumor formation, presented gross photographs of the excised mouse liver/tumor tissues labeled with a 50 μm scale bar. The authors have carefully re-examined the original, unannotated photograph corresponding to Figure 1C. The authors confirm that the 50 μm scale-bar labels shown in the published figure are inappropriate for the gross photographs of the excised mouse liver/tumor specimens.

The original gross photographs were acquired with a camera and did not include a ruler, a calibration scale, or any other object of known dimensions. Consequently, a reliable physical scale in μm or mm cannot be retrospectively established from the photographs themselves. Upon reviewing the original images and the published figure, the authors determined that the 50 μm designation was inadvertently introduced during figure preparation and does not represent a calibrated measurement of the gross specimens.

To ensure that the authors do not provide an unsupported or potentially inaccurate scale, they have revised the figure to remove the 50 μm scale-bar labels from Figure 1C. The purpose of Figure 1C is to illustrate the gross appearance and tumor formation, while the quantitative assessment of tumor burden is presented independently through tumor weight and tumor volume in Figures 1D and 1E. The authors would also like to clarify that the 50 μm scale bars in Figure 1F are appropriate for the H&E-stained histological sections and are unrelated to the scale-bar issue in Figure 1C. Importantly, this issue concerns only the scale-bar annotation in the gross photographic panel and does not affect the underlying experimental data, quantitative measurements, statistical analyses, or conclusions of the published article.

**Figure:**
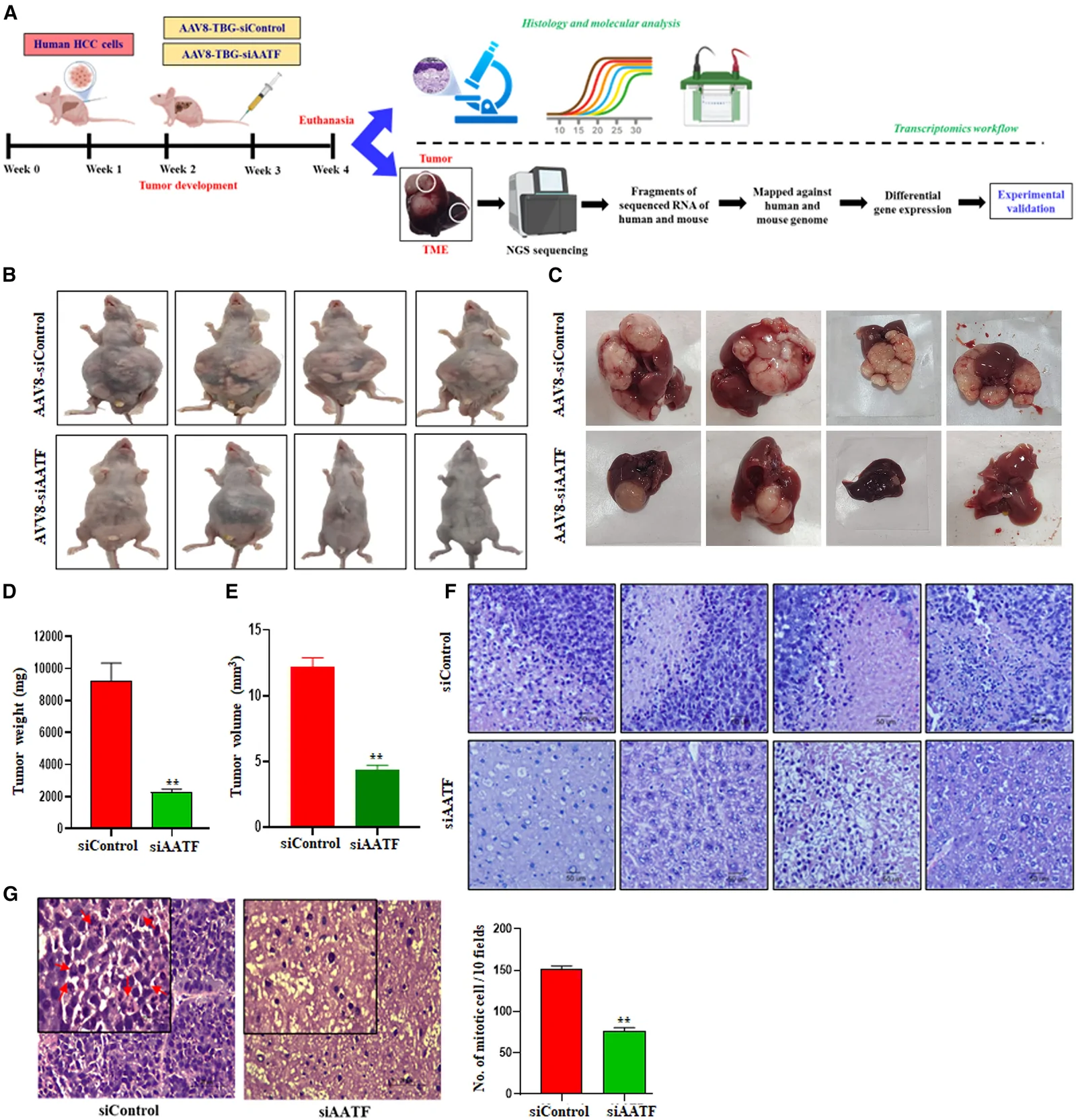
Figure 1. Silencing of AATF reduced tumor burden in the orthotopic xenograft model of HCC (corrected)

**Figure:**
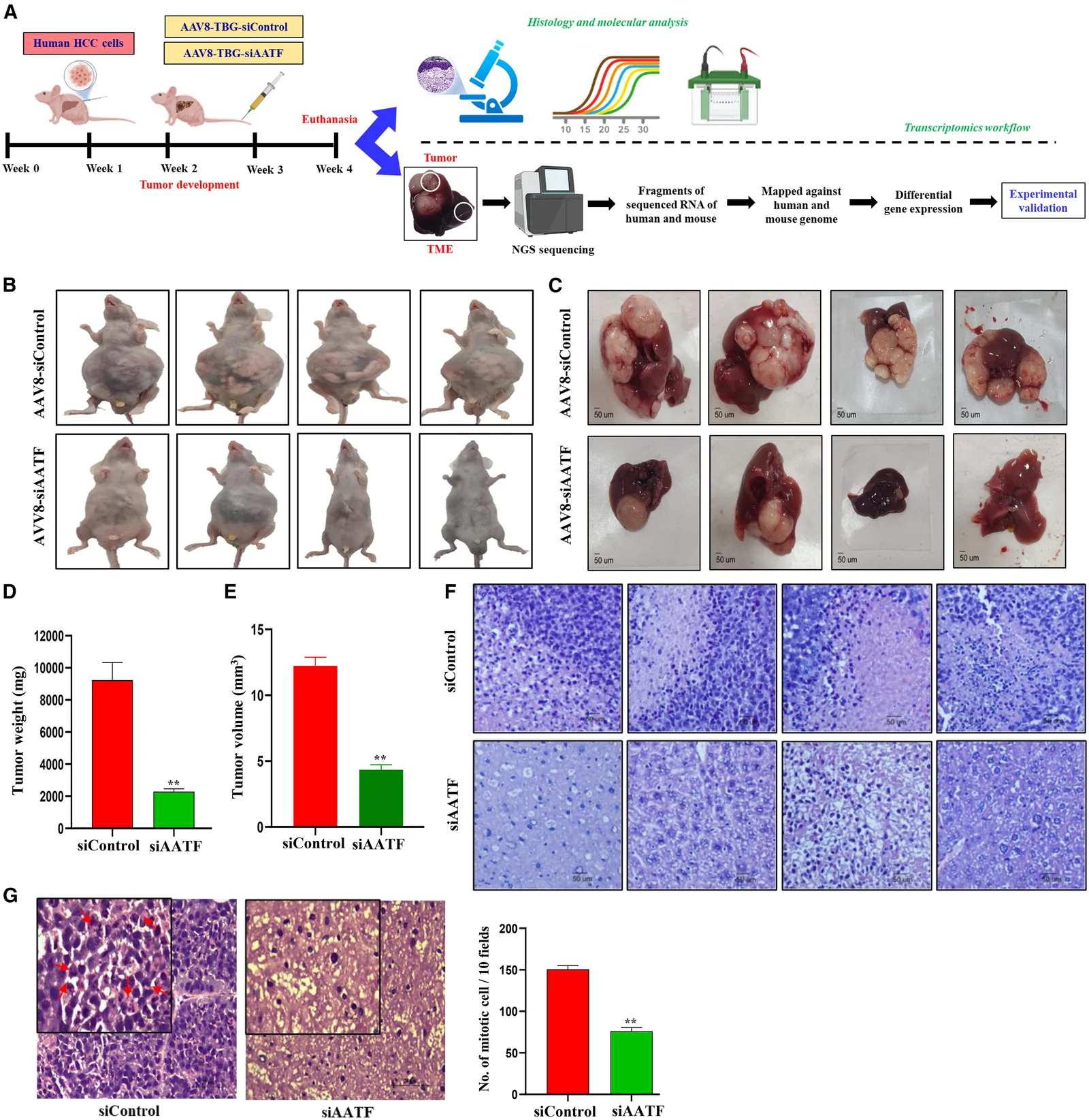
Figure 1. Silencing of AATF reduced tumor burden in the orthotopic xenograft model of HCC (original)

