# Supplementary material for: Targeting AATF reprograms the tumor microenvironment and suppresses hepatocellular carcinoma via MIR100HG-TGF-β signaling
Source: Mol Ther Oncol. 2026 Jun 6;34(3):201259. doi: 10.1016/j.omton.2026.201259 (PMC13334402; doi:10.1016/j.omton.2026.201259)
Supplement: Document S1. Figures S1–S8 and Table S1 [file mmc1.pdf]

OMTON, Volume *34*

Supplemental information

Targeting AATF reprograms the tumor microenvironment and suppresses hepatocellular carcinoma via MIR100HG-TGF-β signaling

Diwakar Suresh, Akshatha N. Srinivas, Bharathwaaj Gunaseelan, S.A. Amith Bharadwaj, Manju Moorthy, Gopalkrishna Ramaswamy, Suchitha Satish, Prashant Vishwanath, Prasanna Kumar Santhekadur, Saravana Babu Chidambaram, and Divya P. Kumar

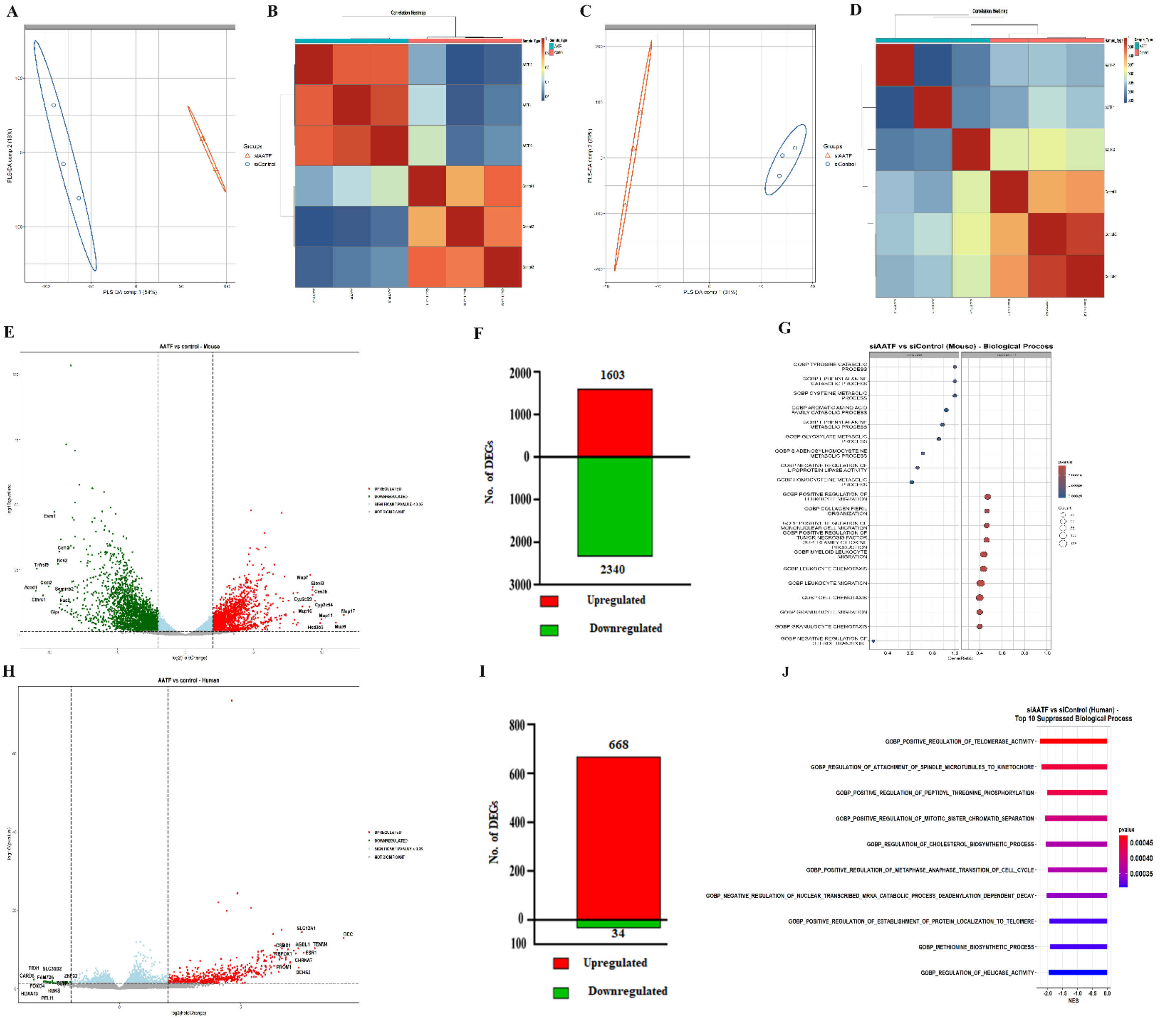

Figure S1. Differential gene expression in tumor and TME following AAV8-TBG-siAATF treatment in an orthotopic xenograft model of HCC

(A) PLS-DA plot, (B) sample correlation plot for TME, (C) PLS-DA plot, and (D) sample correlation plot for tumor showing clear separation between groups, (E) Volcano plot for TME displaying differentially expressed genes. Red dots indicate gene upregulation, green dots indicate downregulation, and grey dots are unchanged ($p<0.05$, log fold≥ 2). (F) Number of genes up- and downregulated in TME transcriptome, (G) Top 10 up- and downregulated GO-BP in siAATF versus siControl group in TME, (H) Volcano plot for tumor transcriptome illustrating DEGs with red dots for upregulation, green for downregulation, and grey dots unchanged ($p<0.05$, log fold≥ 2). (I) Number of genes up- and downregulated in tumor transcriptome, (J) Top 10 downregulated GO-BP in siAATF versus siControl group in tumor. AAV, adeno-associated virus; AATF, apoptosis antagonizing transcription factor; TME, tumor microenvironment; PLS-DA, partial least squares discriminant analysis; GO, gene ontology; BP, biological processes.

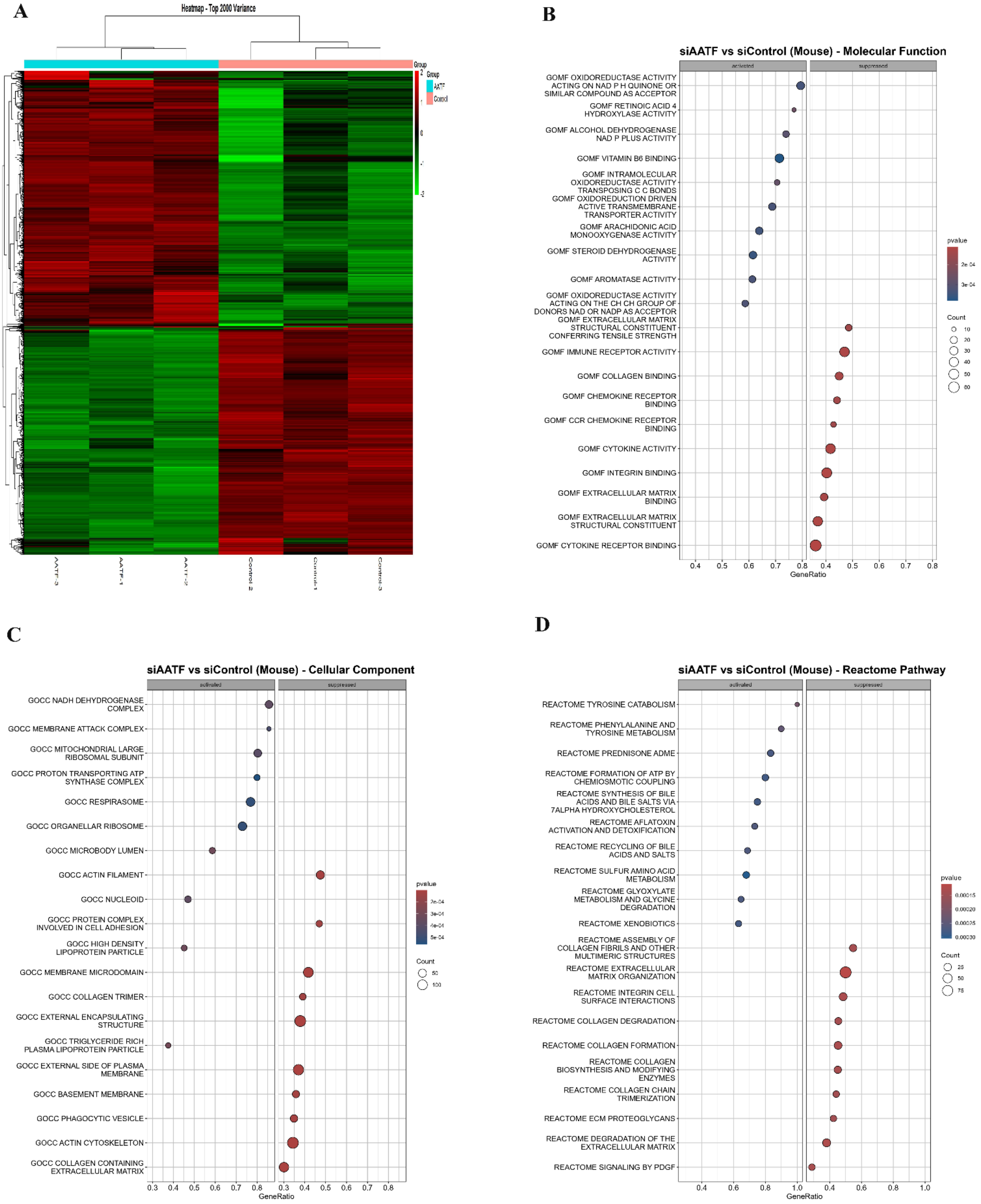

Figure S2. Hierarchical clustering and pathway enrichment analysis of TME in siAATF versus siControl groups

(A) Hierarchical clustering of siAATF versus siControl groups in TME. The top 10 upregulated and downregulated terms in (B) GO-MF, (C) GO-CC, and (D) Reactome pathways in siAATF versus siControl groups in TME. GO, gene ontology; MF, molecular function; CC, cellular component.

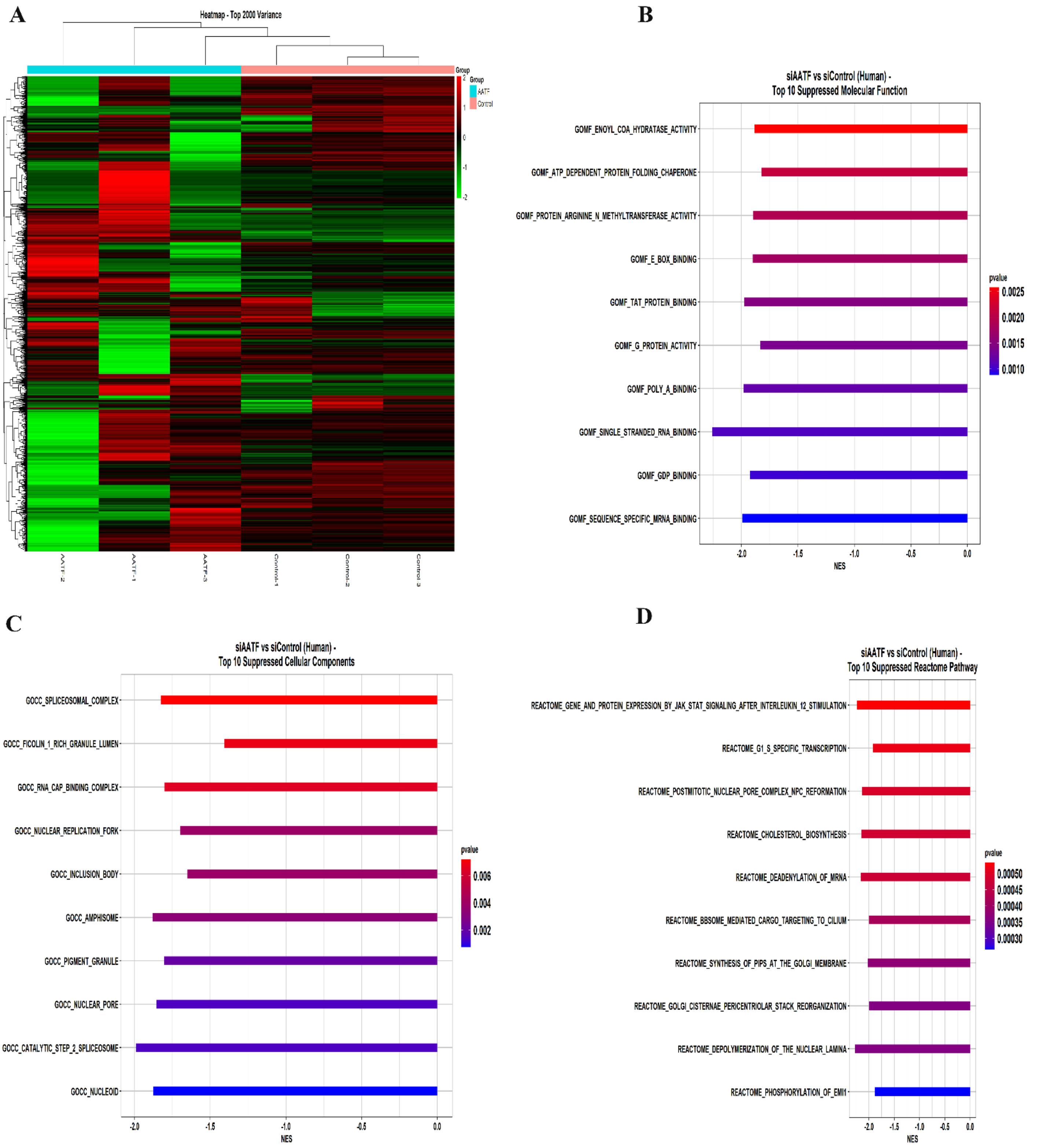

Figure S3. Hierarchical clustering and pathway enrichment analysis of <u>tumors</u> in siAATF versus siControl groups

(A) Hierarchical clustering of siAATF versus siControl groups in the tumor. The top 10 downregulated terms in (B) GO-MF, (C) GO-CC, and (D) Reactome pathways in siAATF versus siControl groups in TME. GO, gene ontology; MF, molecular function; CC, cellular component.

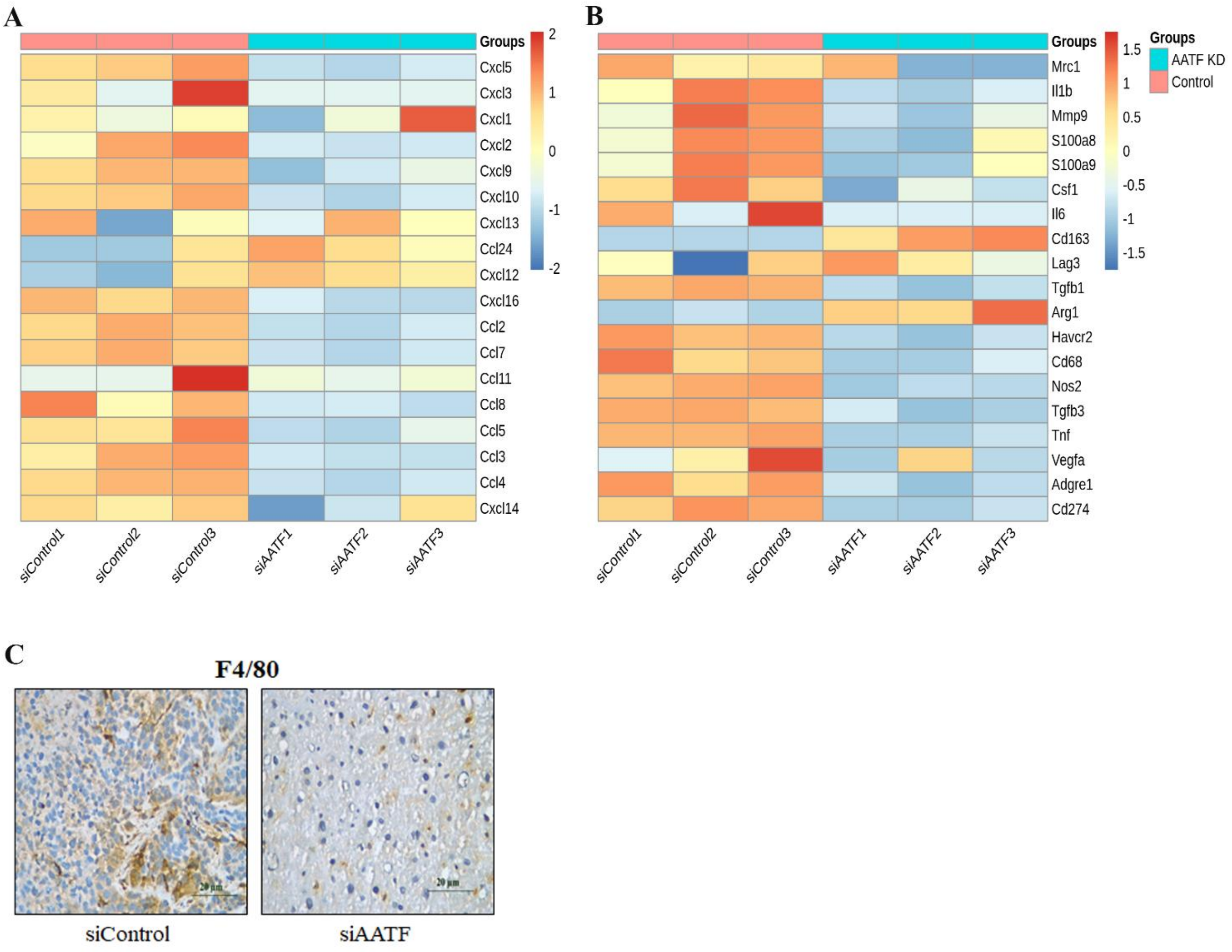

Figure S4. Downregulation of chemokine and immune remodeling genes upon AATF silencing in the TME

Heatmap of chemokines (A) and immune remodeling (B) genes in siAATF versus siControl groups in TME. (C) Representative immunostaining images of F4/80 in tissues from siControl and siAATF mice (400x magnification; scale bar = 20 μm).

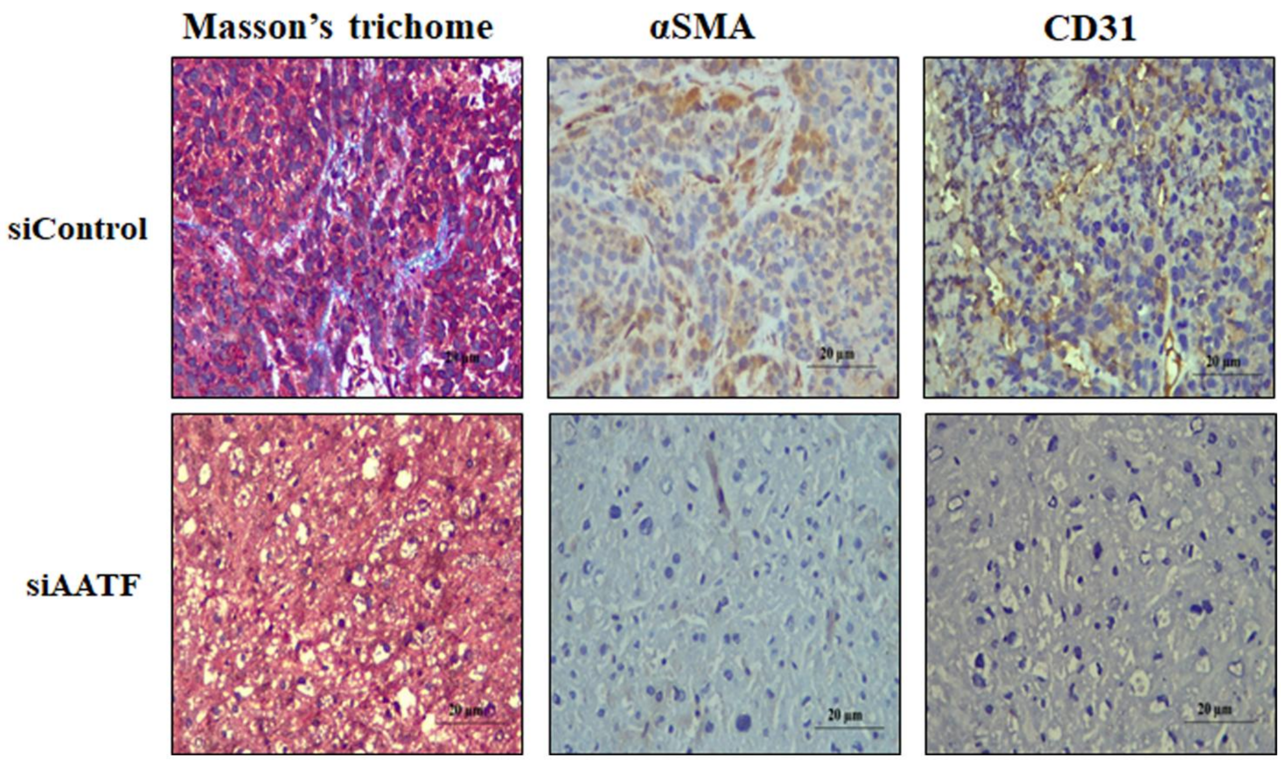

Figure S5. AATF silencing disrupts extracellular matrix remodeling and angiogenesis in the tumor microenvironment

Representative microscopic images of (A) Masson's Trichrome staining, (B) α-SMA immunostaining, and (C) CD31 immunostaining in liver sections from siControl and siAATF mice (400x magnification; scale bar = 20 µm). α-SMA, alpha-smooth muscle actin; CD31, cluster of differentiation 31.

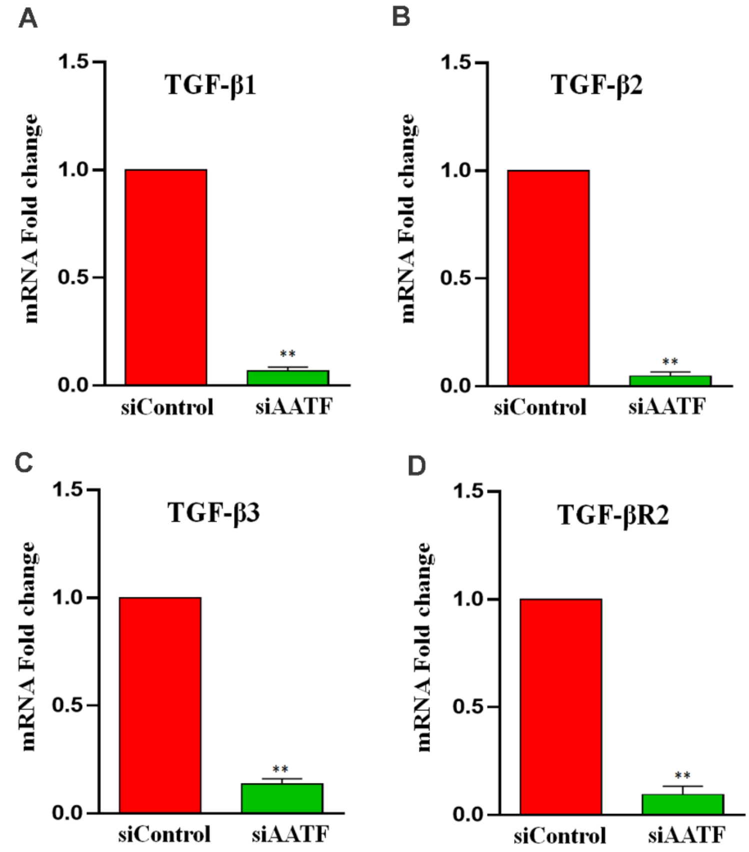

Figure S6. Expression profiling of TGF-β ligands and receptors

mRNA expression of TGF-β1 (A), TGF-β2 (B), TGF-β3 (C), and TGF-βR21 (D) expressed as fold change. Data are presented as mean ± SEM for n=3 per group, **$p<0.001$ compared to siControl, unpaired t-test. TGF-β, transforming growth factor- beta; TGF-βR, transforming growth factor- beta receptor.

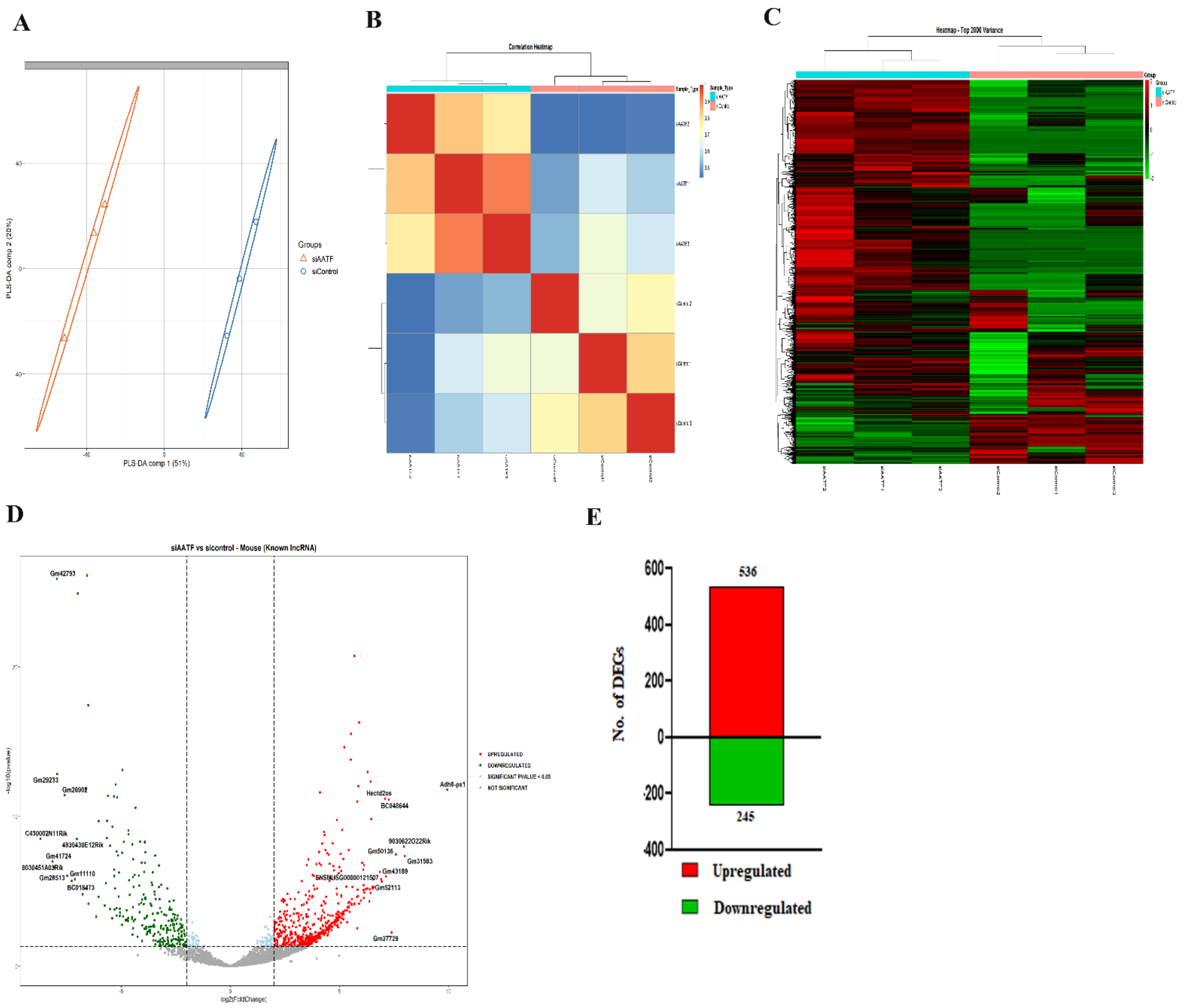

Figure S7. Transcriptomic analysis of mouse lncRNAs in the tumor microenvironment

(A) PLS-DA plot, (B) sample correlation plot, (C) hierarchical clustering of mouse lncRNAs, (D) Volcano plot showing differentially expressed lncRNAs. Red dots indicate gene upregulation, green dots indicate gene downregulation, and grey dots are unchanged (p<0.05, log fold≥ 2), and (E) number of lncRNAs upregulated and downregulated in TME.

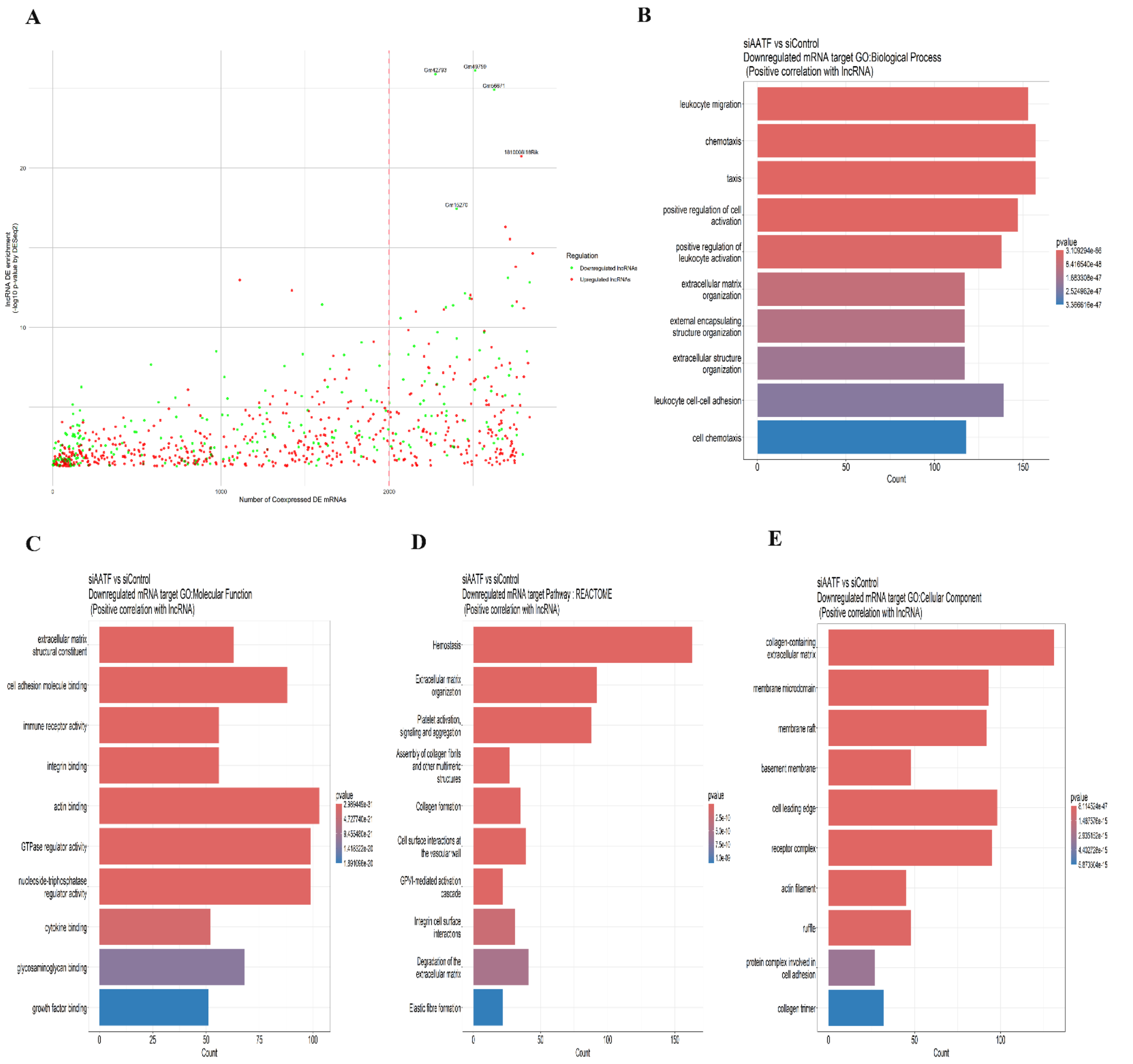

Figure S8. Correlation and functional enrichment analysis of downregulated lncRNA and mRNA

(A) Pearson's correlation analysis of co-expressed lncRNA and mRNA, (B) GO-BP, (C) GO-MF, (D) GO-CC, and (E) Reactome pathway analysis for positively correlated downregulated lncRNA-mRNA.

Table S1. List of primer sequences used in qRT-PCR in the study.

Primer	Sequence
Mouse AATF	**F:** GAGTGATGATGCCAGGACGGA<br>**R:** ACTGTCACTTCCCACGGTCTG
Mouse IL-1β	**F:** TGCCACCTTTTGACAGTGATGA<br>**R:** TGATGTGCTGCTGCGAGATTTG
Mouse IL-6	**F:** GGAGCCCACCAAGAACGATA<br>**R:** AACTGGATGGAAGTCTCTTGC
Mouse TNF-α	**F:** TAGCCCACGTCGTAGCAAACC<br>**R:** CTTTGAGATCCATGCCGTTGGC
Mouse Col1A1	**F:** TGACTGGAAGAGCGGAGAGTA<br>**R:** AGACGGCTGAGTAGGGAACA
Mouse Col3A1	**F:** GACCTAAGGGCGAAGATGGC<br>**R:** GAAGCCACTAGGACCCCTTTC
Mouse α-SMA	**F:** CTACTGCCGAGCGTGAGATTGT<br>**R:** CCCGCTGACTCCATCCCAATGA
Mouse TGF-β	**F:** GCTGCATATCGTCCTGTGG<br>**R:** CTTCCATTTCCACATCCGACT
Mouse β Actin	**F:** CAGCCTTCCTTCTTGGGTATGG<br>**R:** CCTGCTTGCTGATCCACATCT
Mouse AFP	**F:** AACCTCCAGGCAACAACCATT<br>**R:** CACTCCTCGTGGATGTGAGC
Mouse CD31	**F:** GTGGAAGTGTCCTCCCTTGA<br>**R:** GGGAGCCTTCCGTTCTAGAGTAT
Mouse MIR100HG	**F:** AGGGAGCGATTGGCAGAAGA<br>**R:** GCTTCCGTGTTGCCGATTTCT
Mouse E-cadherin	**F:** ATCCTCGCCCTGCTGATTCT<br>**R:** GGCTCTTTGACCACCGTTCTC
